# Development and Characterization of Ethylcellulose Oleogels Based on Pumpkin Seed Oil and Rapeseed Oil

**DOI:** 10.3390/gels10060384

**Published:** 2024-06-05

**Authors:** Claudiu-Ștefan Ursachi, Simona Perța-Crișan, Iolanda Tolan, Dorina Rodica Chambre, Bianca-Denisa Chereji, Dumitru Condrat, Florentina-Daniela Munteanu

**Affiliations:** Faculty of Food Engineering, Tourism and Environmental Protection, “Aurel Vlaicu” University of Arad, 310330 Arad, Romania; claudiu.ursachi@uav.ro (C.-Ș.U.); simona.perta-crisan@uav.ro (S.P.-C.); iolanda.tolan@uav.ro (I.T.); dorina.chambre@uav.ro (D.R.C.); bianca.chereji@uav.ro (B.-D.C.); dumitru.condrat@uav.ro (D.C.)

**Keywords:** pumpkin seed oil, rapeseed oil, oleogelator, ethylcellulose, oleogel

## Abstract

In contrast to rapeseed oil, pumpkin seed oil has yet to be well investigated in terms of oleogelation, and, to the best of our knowledge, no study related to the use of ethylcellulose (EC) in the structuring of this oil has been identified in the current scientific literature. Therefore, the present study evaluated several oleogels formulated with EC as the oleogelator in different concentrations of 7% (OG7) and 9% (OG9), based on cold-pressed pumpkin seed oil (PO) and refined rapeseed oil (RO), as well as on mixtures of the two oils in different combinations: PO:RO (3:1) (PRO) and PO:RO (1:1) (RPO). Physicochemical properties such as visual appearance, gel formation time (GFT), oil-binding capacity (OBC), oxidative and thermal stability, and textural characteristics were analyzed. Analysis of variance (ANOVA) and Tukey’s honestly significant difference (HSD) were used in the statistical analysis of the data, with a significance level of *p* < 0.05. EC proved to be an effective structuring agent of the mentioned edible oils; the type of oils and the concentration of oleogelator significantly influenced the characteristics of the obtained oleogels. The 9% EC oleogels exhibited a more rigid structure, with a higher OBC and a reduced GFT. Pumpkin seed oil led to more stable oleogels, while the mixture of pumpkin seed oil with rapeseed oil caused a significant reduction in their mechanical properties and decreased the OBC. After 14 days of storage, all oleogels demonstrated proper oxidative stability within the bounds set by international regulations for edible fats, regardless of the kind of oil and EC concentration. All of the oleogels showed a higher oxidative stability than the oils utilized in their formulation; however, those prepared with cold-pressed pumpkin seed oil indicated a lower level of lipid oxidation among all oleogels. The P-OG9 and PR-OG9 oleogels, which mainly included PO and contained 9% EC, demonstrated the optimum levels of quality in texture, GFT, OBC, and oxidative stability.

## 1. Introduction

Oleogelation is an intensively researched technology that transforms liquid oils into gels without chemically changing them. Over time, two large categories of structuring agents for different oils have been identified, namely lipid components such as fatty acids and fatty acid esters, glycerolipids, glycerophospholipids, sterol lipids, and fatty alcohols, and non-lipid components such as some proteins and polysaccharides [1]. EC is a derivative of cellulose recognized as a food-grade non-toxic additive (E462) used as an emulsifier, thickener, or stabilizer in various food products [2]. EC is a clean-label ingredient that can be used to reduce the use of synthetic stabilizers and emulsifiers [3]. Its properties can be customized to meet specific application requirements, allowing customized solutions for food products and processing conditions [4]. EC is a versatile polymer used in the food industry for high-temperature oleogels. It is one of the few polymers that can be used to convert oils into oleogels through the direct dispersion method [5], although some other polysaccharides with similar behavior of forming three-dimensional networks that entrap oils have been successfully studied: agar, xanthan gum, chitin and chitosan, and pectins [1]. Due to its semi-crystalline structure and hydrophobic character, EC can form oleogels characterized by a solid and stable structure, with a high nutritional value induced by the fatty acid content of the used vegetable oils [6]. Their textural and rheological properties have been proven to be associated with the oil type used, the concentration and quality of the oleogelator and the conditions of obtaining it [6,7]. However, a significant inconvenience in this sense is the high-temperature heat treatment necessary for ensuring the dissolution of EC into oils, due to its high glass transition temperature (125–140 °C) [8,9] that can accelerate lipid oxidation and reduce the oxidative stability of oleogels [5]. Its thermal stability, compatibility with oils, and viscosity control make it ideal for various food applications [10], allowing for incorporation into baked goods, dairy products, and spreads without significant modifications [11]. Its good oil-binding capacity stabilizes the oleogels’ structure and prevents oil separation, contributing to food products’ stability and shelf life [12].

In recent years, pumpkin seed oil, extracted from the seeds of pumpkins by mechanical pressing, has gained attention for its nutritional profile and associated health benefits [13]. This oil is a rich source of nutrients, including essential fatty acids (mainly oleic and linoleic acids), sterols (especially β-sitosterol, stigmasterol, and ∆−5- and ∆−7 sterols), vitamin E, and minerals (such as zinc and magnesium) [14,15]. The high contents of bioactive components in pumpkin seed oil are linked to several potential health benefits, such as supporting heart health, promoting prostate health, reducing inflammation, and improving skin and hair health [16].

Rapeseed oil is extracted from the seeds of the rapeseed plant (Brassica napus or Brassica rapa) [17]. There are two types of rapeseed oil: canola oil and traditional rapeseed oil. Canola oil is a variety of rapeseed characterized by reduced levels of erucic acid and glucosinolates, which make it safer for consumption. Traditional rapeseed oil, also called “virgin rapeseed oil”, is obtained from non-modified rapeseed and may have higher levels of erucic acid and glucosinolates [18]. Rapeseed oil is also considered healthy due to its fatty acid composition, low content of saturated fats, and high quantities of monounsaturated and polyunsaturated fats, including omega-3 and omega-6 fatty acids. Additionally, it contains vitamin E, which acts as an antioxidant [19].

With regard to the organoleptic properties of the mentioned oils, pumpkin seed oil possesses a complex, distinctly nutty and woody flavor and a deep green color, which may constitute drawbacks in obtaining oleogels further used as fat replacers. In contrast, rapeseed oil has a mild, neutral flavor and color. To our knowledge, few studies can be found in the current scientific literature on the development of pumpkin seed oil-based oleogels [7,20], and only by using various waxes as oleogelators, while their applicability in food products is noted only in margarine and creams [21,22]. One factor would be the specific sensory properties of pumpkin seed oil, which could be an obstacle to producing oleogels utilized as alternatives to fat.

In contrast, rapeseed oil has been intensively investigated in terms of oleogelation with different oleogelators such as waxes, shellac, ethylcellulose, and monoglycerides. Many studies highlight the use of rapeseed oil-based oleogels in various food products like spreads, cakes, biscuits, and patties [23,24,25,26].

Considering all the presented aspects, the present study aimed to formulate novel EC oleogels based on pumpkin seed oil, used alone or associated in different proportions with rapeseed oil, to diminish its sensory inconveniences and to obtain oleogels with both acceptable organoleptic characteristics and optimal physicochemical properties. The most suitable formulas were searched for to replace saturated fats in foods.

## 2. Results and Discussion

### 2.1. Visual Appearance

**Figure 1:**
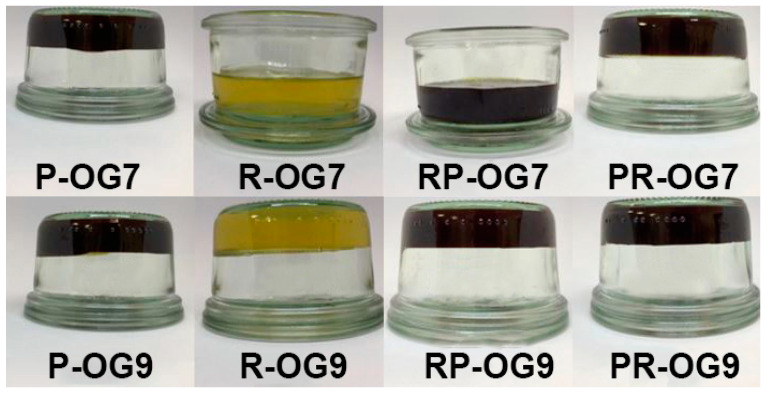
Visual appearance of EC oleogel samples.

The visual appearance of the developed EC oleogels is presented in Figure 1. As shown by other authors [5,27], our findings also suggest that the concentration of EC substantially impacts the stability, structure, and texture of the oleogels. For the samples with 7% EC, the different combinations of oils resulted in various levels of gel stability. Only two formulations formed self-standing oleogels, entrapping the liquid oil in a three-dimensional network. According to Figure 1, among the four OG7 samples, only P-OG7 and PR-OG7 exhibited a solid gel structure, while R-OG7 and RP-OG7 did not solidify and remained in a semi-liquid state.

In contrast, the 9% EC concentration oleogels consistently produced stable and solid gel structures in all formulations. The color of the oleogels was defined by the type of oil rather than the concentration of EC. Overall, these observations provide only a brief understanding of how various oil combinations and EC concentrations impact the visual properties of oleogels.

### 2.2. Gel Formation Time

Table 1 shows the gel formation time (GFT) for the developed oleogels, which exhibited variations due to the differences recorded in their composition. Except for the two samples, R-OG7 and RP-OG7, which did not form gel and remained in a fluid or semi-fluid state, all other samples indicated a rapid gelation that occurred in a minimal period, typically between 2.00 and 3.50 min. According to the presented data, increasing the EC concentration from 7 to 9% reduced the GFT from 3.10 min to 2.00 min for P-OG and from 3.50 min to 2.15 min for PR-OG. The results are in accordance with other studies, which reported that increasing the level of oleogelator caused a decrease in GFT [28]. As seen in Table 1, the type of oil was found to have a significant effect on the gelation and mechanical properties of EC oleogels. There must be sufficient oleogelator to achieve the so-called critical gelation concentration [29], capable of forming a stable three-dimensional network. Therefore, R-OG7 and PR-O G7 gels could not be obtained. EC gel formation also involves polymer–solvent and solvent–solvent interactions. Gravelle et al. [30] consider that oils with a high level of unsaturation generate a higher solvent–polymer interaction, which could significantly impact gel strength. PO and RO have considerably different compositions of polyunsaturated fatty acids (PUFAs) [17,31], and this can be considered an explanation for the gelling behavior of the two oils, as shown in Section 2.6.

### 2.3. Oil-Binding Capacity

**Figure 2:**
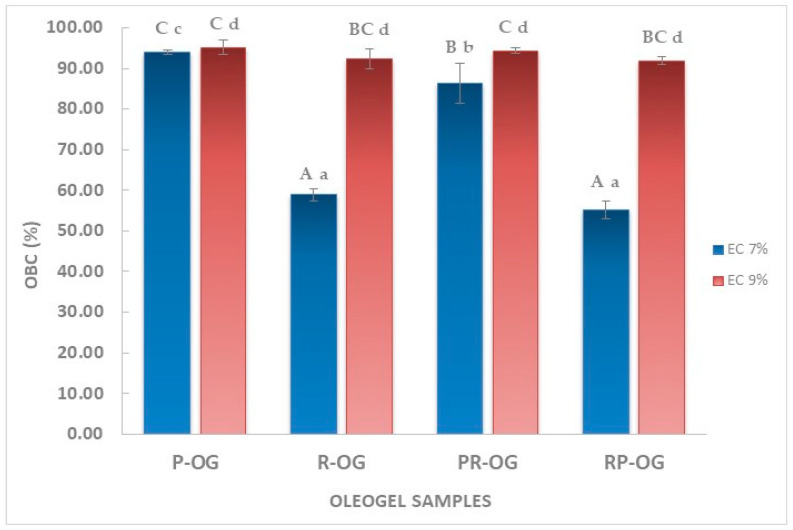
Oil-binding capacity of oleogel samples. Different capital letters indicate significant differences within oleogel samples (*p* < 0.05). Different small letters indicate significant differences within oleogel samples with same EC concentration (*p* < 0.05).

OBC is an important quality parameter of oleogels and indicates the gel structure’s capability to keep the liquid phase entrapped [32]. The results for the OBC of the prepared oleogel samples are presented in Figure 2. In accordance with other studies [33,34] that compared samples with different EC concentrations, a higher oil retention capacity of oleogels was observed with increasing oleogelator concentration. The same behavior was also observed in our study; by raising the EC concentration from 7% to 9%, the OBC increased for all samples, ranging between 1.14% for P-OG and 36.69% for RP-OG. It should be noted that, in the case of oleogels obtained exclusively from cold-pressed pumpkin oil, the differences recorded for OBC values are insignificant, regardless of the EC concentration (*p* < 0.05). For all other oleogel samples, where rapeseed oil was used in different proportions (R-OG, PR-OG, and RP-OG), the differences in OBC were significant at different EC concentrations, 7% and 9%. Samples R-OG7 and RP-OG7 had the lowest OBC values, with no significant differences between them (*p* < 0.05). Therefore, it was noticed that the oil-binding capacity decreased significantly in the case of oleogels, with rapeseed oil in the composition at a concentration of 7% EC, while, by increasing it to 9%, this inconvenience is eliminated. Regardless of the oil type used, oleogels with 9% EC showed a proper capability for oil binding and forming continuous and stable networks. Moreover, regardless of EC concentration, pumpkin seed oil-based oleogels were the most stable, with reduced amounts of oil released.

### 2.4. Attenuated Total Reflectance–Fourier Transform Infrared

**Figure 3:**
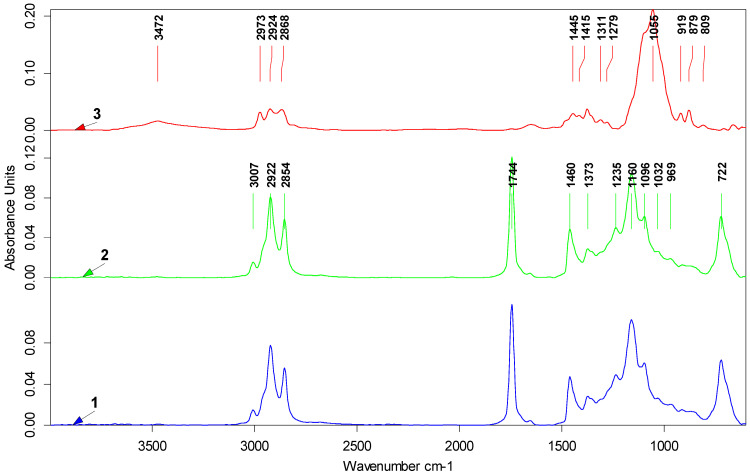
ATR-FTIR spectra of 1—PO; 2—RO; 3—EC.

**Figure 4:**
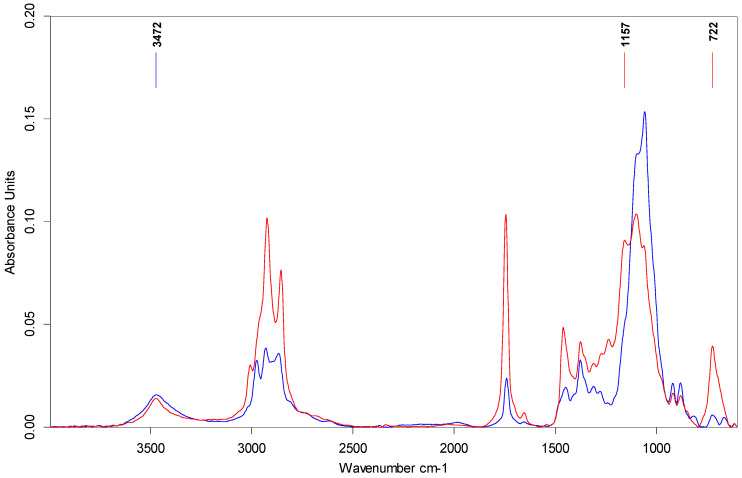
ATR-FTIR spectra of RP-OG7 and RP-OG9 oleogels.

**Figure 5:**
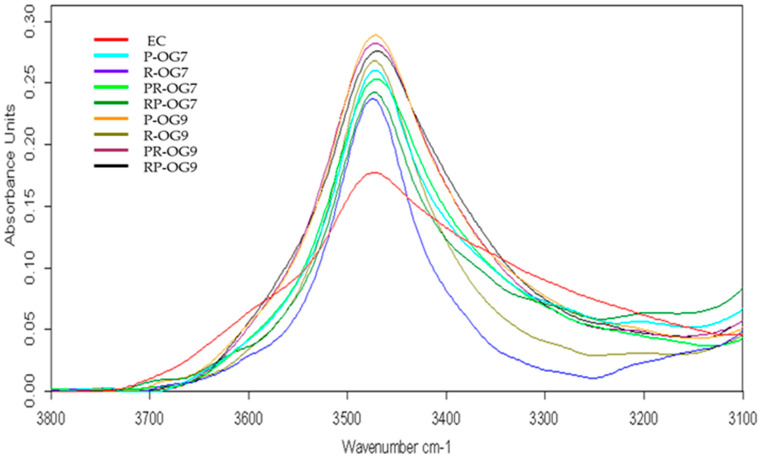
ATR-FTIR spectra in 3800–3250 cm^−1^ range of EC and oleogel samples.

Attenuated Total Reflectance–Fourier Transform Infrared (ATR-FTIR) spectroscopy was employed to study the polymer–polymer interactions occurring among the macromolecules of the EC structuring agents or the solvent–polymer interactions between EC and vegetable oils. Figure 3 depicts the ATR-FTIR spectra recorded for PO, RO, and EC samples. The spectra of the PO and RO exhibit a similar profile, characterized by typical vibrational bands for vegetable lipids, as was also observed in other studies [35,36,37,38]. The symmetric (ν_sym_) and asymmetric (ν_asym_) stretching vibrations of the C-H bond from CH_2_ and CH_3_ groups were detected within the 3100–2800 cm^−1^ range. Specifically, peaks were observed at 3007 cm^−1^ (ν_sym_ for =C-H cis-, 2922 cm^−1^ (ν_sym_) for -CH_2_, and 2853 cm^−1^ (ν_asym_) for CH_2_, as also reported by Yuzhen et al. [37]). Another distinct band was observed at 1744 cm^−1^, attributed to the stretching vibration of the ester’s carbonyl group (C=O). Within the fingerprint range (1500–1000 cm^−1^), the detected peaks are located at 1460 cm^−1^ (CH_2_ bending), 1373 cm^−1^ (CH_2_ bending), 1235 cm^−1^ (C-O stretching), 1160 cm^−1^ (C-O stretching and -CH_2_ bending), 1096 cm^−1^ (C-O stretching), and 1032 cm^−1^ (C-O stretching) [35,36,39]. A peak indicates the presence of trans isomers at 969 cm^−1^, attributed to the out-of-plane bending of -HC=CH- (trans-). Additionally, the rocking vibration of the -(CH_2_)n, -HC=CH- (cis-) groups was recorded as an intense peak at 722 cm^−1^ [40]. The EC spectrum is shown in the 3750–3100 cm^−1^ wavenumber range, a broad band with low intensity (~3472 cm^−1^), which is attributed by Lin et al. [41] to the stretching vibration of the unsubstituted -OH groups of cellulose. The characteristic bands of C-H stretching vibrations from CH_2_ and CH_3_ groups were recorded at 2973 cm^−1^ (ν_asym_ CH_3_), 2924 cm^−1^ (ν_asym_ CH_2_), and 2868 cm^−1^ (ν_sym_ CH_3_). Another intense band was recorded in the 1224–940 cm^−1^ range with maximum absorbance values of 1055 cm^−1^, which was attributed to the stretching vibration of C-O-C from the pyranose ring and aliphatic ether groups. As reported elsewhere, the 919 cm^−1^ band can be attributed to the C-O-C vibration of β-glycosidic linkage between the glucose units [42]. The characteristic ATR-FTIR spectra of prepared oleogels are exemplified in Figure 4 for RP-OG7 and RP-OG9 samples. The other oleogel spectra looked similar to those in Figure 4 and showed the typical absorption bands for EC, PO, and RO, respectively. According to Silva et al. [33], the FTIR spectra of the oleogels are dominated by the characteristic bands of the oils used for formulation, these being the main components. In the case of our samples, this behavior was observed for oleogels with 7% EC. By increasing the concentration of EC to 9%, the ATR-FTIR spectra of the samples became dominated by the oleogelator, and the oils-specific bands decreased in intensity, as seen in Figure 4. Compared to the EC spectrum, in the case of oleogels, a more symmetrical and intense broadband was recorded at ~3472 cm^−1^, attributed in the literature to the stretching vibration of the -OH group associated intra- and intermolecularly through hydrogen bridges [43]. When heated and dissolved in vegetable oils, the EC semi-crystalline polymer chains acquire a degree of freedom, which allows them to rearrange in space, resulting in a flexible polymer [41]. The liquid oil is embedded in the cross-linked network formed on cooling, as previously shown by others [44,45]. Under these conditions, additional hydrogen bridges can form between the un-ethylated OH groups of the cellulose. As a consequence, the shape of this band is changed compared to EC, mainly due to polymer–polymer interactions that lead to the formation of the three-dimensional network of oleogels [46]. In addition, the intensity of this band can also be influenced by the polymer–solvent interactions between the OH groups from EC and some compounds naturally present in unrefined PO [45], as seen in Figure 5.

Also, in the case of oleogels with 9% EC, a decrease in the intensities of the bands specific to oils was observed (C-H stretching, C=O stretching, C-H bending, CO stretching, rocking C-H, and C-C-H bending vibrations) and there was an increase in the intensity of the specific EC band located at 1055 cm^−1^. The band at 1160 cm^−1^, due to CH_2_ bending from the oil’s spectra, in gels with 7% EC, shifted at 1155 cm^−1^ and decreased significantly in intensity, while in gels with 9% EC, it was recorded as a shoulder. Silva et al. [33] reported similar behavior, attributed to the establishment of polymer–solvent interactions between oils and EC in the oleogel network. As an effect of these interactions, the fatty acids from triacylglycerols were more strongly anchored by binding in the three-dimensional matrix of the gels, having a reduced steric mobility, which led to a decrease in the intensity of in-plane and out-of-plane bending vibrations (1157 cm^−1^ and 722 cm^−1^) characteristic for -CH_2_ groups, previously also shown by Lupi et al. [47].

### 2.5. Oxidative Stability

Because the obtaining of oleogels using the ethylcellulose as an oleogelator requires a high-temperature thermal treatment, which for the polymer–oil mixture may have a negative effect, then the evaluation of lipid oxidation is an important quality parameter, mainly due to the presence of polyunsaturated fatty acids from PO and RO.

#### 2.5.1. Peroxide Value

**Figure 6:**
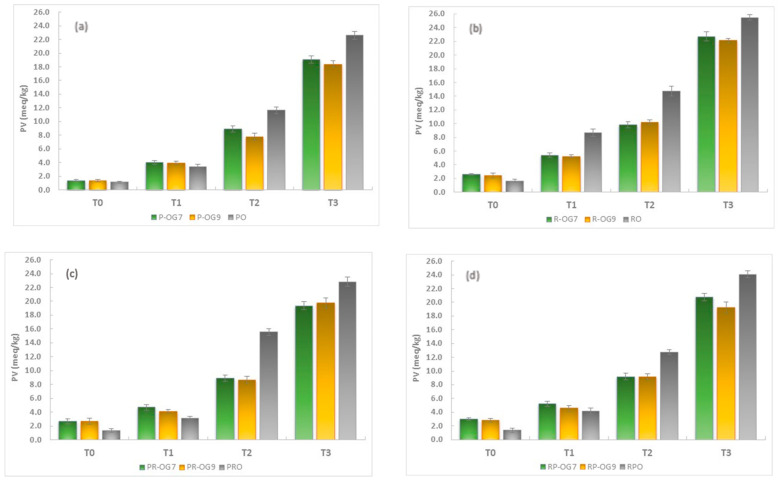
Evolution of PV of oils and EC oleogels during storage: (**a**) PO and P-OGs, (**b**) RO and R-OGs, (**c**) PO:RO (3:1) and PR-OGs, (**d**) PO:RO (1:1) and RP-OGs.

The peroxide value (PV) was used to measure the oxidation level of oleogels as evidence of primary oxidation after processing and during storage. In the primary stages of oxidation, fatty acids react with oxygen to form odorless compounds such as peroxides [48,49]. The recorded PVs for oils and oleogels, after processing and during storage, are shown in Figure 6. The initial PV of the oils and oleogels (T0) recorded values between 1.150 ± 0.139 meq O_2_/kg oil (PO) and 2.970 ± 0.192 meq O_2_/kg oil (RP-OG7) and suggest that primary oxidation levels after processing are low (<10 meq O_2_/kg oil). All the oleogel samples showed a higher PV compared to the fresh oil before storage, and it could be attributed to the fact that the oils were heated at high temperatures during the oleogel preparation. After 7 days of storage (T1), a linear increase in PV can be observed for all the samples. The results showed that PO (3.380 ± 0.354 meq O_2_/kg oil) was the most stable oil sample, while in terms of oleogels, the P-OG9 (3.970 ± 0.277 meq O_2_/kg oil) was the most stable and R-OG7 (5.420 ± 0.304 meq O_2_/kg oil) the least stable samples. These values are considered satisfactory according to the international regulations for edible oils (less than 10 meq O_2_/kg oil) [50]. PV recorded a significant increasing trend in all samples after 14 days of storage (T2), indicating an accelerated degradation process. Except for R-OG9 (10.18 ± 0.344 meq O_2_/kg oil), PV for all other oleogels was less than the acceptability level. PV continued to increase for all samples during storage, and after 21 days (T3), it reached maximum values, exceeding the acceptability limit due to the formation of advanced oxidation products.

Since the study’s main objective was to analyze the influence of different factors on the oleogels properties, we consider that EC concentration and oil type contribute significantly to the oxidative stability of the oleogels. Regarding the EC concentration, the results showed lower oxidation for oleogels with a concentration of 9% compared to those with 7%, which can be explained by the immobilization of oils in the network of the oleogelator. Due to the higher viscosity and stability of the 9% EC oleogel network, the access to pro-oxidizing factors (light, heat, oxygen, and metal ions) was limited. Although the preparation of EC-based oleogels resulted in an increased PV compared to fresh oils due to the high temperature applied for processing (155 °C), their PV values were lower than those of the oils at the end of the storage period. These results showed the importance of gel networks in inhibiting oil oxidation, as the compact structure of oleogels considerably reduced the ability of oxygen to penetrate and diffuse into the samples.

Regarding the type of oil used, the results showed that, under the same storage conditions, after 21 days, P-OG9 was the most stable sample (18.380 ± 0.501 meq O_2_/kg oil), while R-OG7 was the most unstable oleogel (22.700 ± 0.666 meq O_2_/kg oil). These findings showed that the oxidation process differed depending on the oil type. Our data indicated that the pumpkin seed oil-based oleogels showed the highest oxidative stability, which can be explained by the presence of natural antioxidants (tocopherols, phytosterols, polyphenols, and flavonoids) in unrefined oils compared to refined ones [51]. These observations agree with the conclusions of other studies [52,53,54]. On the other hand, oils possessing higher levels of saturated fatty acids and lower polarities can improve the oxidative stability of oils when they are entrapped in the internal structure of oleogels [10,55]. Several authors [31,56,57] reported that pumpkin seed oil contains approximately 20% saturated fatty acids and 80% unsaturated fatty acids, while, in rapeseed oil, the content of saturated fatty acids is only 6–7% and 93–94% of unsaturated fatty acids, respectively.

Furthermore, in contrast with rapeseed oil, pumpkin seed oil has a higher polarity due to its diversified composition of fatty acids and to the presence of some bioactive compounds such as phytosterols, tocopherols or carotenoids [51], which can affect the structure of EC network and also influence the oxidation behavior of the oleogels [7]. Regarding the mixtures of the two oils, our results confirmed the previous hypotheses, showing that the presence of pumpkin oil increased the oxidative stability of the oleogels. The PV values of the PO:RO 3:1 mixture were higher than those of PO, but lower than the PO:RO 1:1 mixture.

#### 2.5.2. TBARS

**Figure 7:**
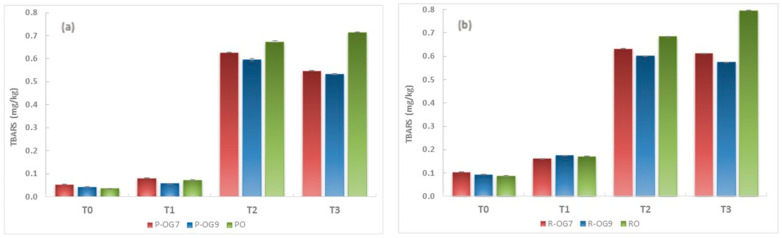

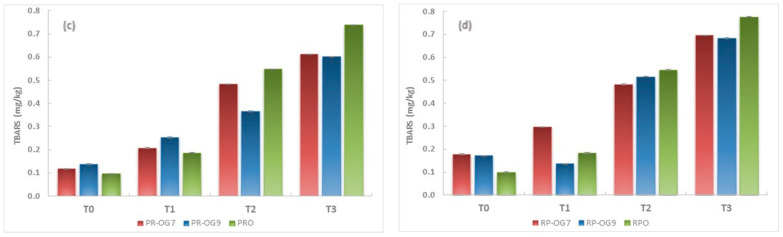
Evolution of TBARS value of oils and EC oleogels during storage: (**a**) PO and P-OGs, (**b**) RO and R-OGs, (**c**) PO:RO (3:1) and PR-OGs, (**d**) PO:RO (1:1) and RP-OGs.

In parallel with PV determination, the TBARS values, a widely used indicator of secondary oxidation products, were also monitored in this study. Figure 7 shows the changes in TBARS values of the samples as a function of storage time at 60 °C. It can be noticed that, as well as PV, TBARS values increased during storage. Initial TBARS (T0) ranged between 0.035 ± 0.002 mg/kg and 0.099 ± 0.002 mg/kg for the oil samples and between 0.042 ± 0.002 mg/kg and 0.178 ± 0.002 mg/kg for the oleogel samples, with no critical changes observed in TBARS values after their preparation. During storage, all samples’ TBARS values increased gradually due to secondary lipid oxidation. However, it can be noticed that the increasing trend in oleogel samples was slower than in oils due to the structure of the oleogelator network, which slowed the transfer of oxygen radicals and prevented the generation of secondary oxidation products. Furthermore, the oxidation degree of the oleogel samples decreased with increased EC concentration from 7% to 9%, since the EC network structure became denser and formed physical solid barriers. Similar results were obtained by Oh et al. [58], who suggested that the oxidative stability of oleogels obtained with hydroxypropyl methylcellulose as an oleogelator was dependent on its concentration.

### 2.6. Texture Analysis

The firmness of oleogels is one of the most important textural properties for determining the possibility of using them in various food formulations [59]. It is well known that the texture of edible oil-based EC oleogels is dependent on a variety of parameters, such as the oil type through the unsaturation level and fatty acids profile, the working conditions, and the molecular weight, viscosity, and concentration of EC [5,27]. Although the influence of oil composition on the texture of oleogels is recognized, a coherent study about how the mixture of different oils affects gel strength has yet to be published. Table 2 presents the texture parameters obtained for the various EC oleogels measured at 5 °C.

Interesting results were obtained regarding the texture of the studied oleogels. As can be observed, the texture of the samples was significantly influenced by the type of oil and the concentration of the oleogelator (EC). In accordance with another study [6], the results showed a strong correlation between oleogel hardness and polymer concentration. Regardless of the type of solvent, under the same conditions, raising the EC concentration from 7% to 9% led to significant increases in firmness and adhesiveness for all samples, suggesting better mechanical properties due to the dense structural organization of the oleogels. The significant differences in texture parameters between OG samples with different EC concentrations (R-OG7 and R-OG9, RP-OG7 and RP-OG9, respectively) may be caused by the internal morphology of EC [6,27]. The authors believe the oleogel pore size is closely related to the oleogelator concentration. The reduction in the pore diameter by increasing the oleogelator concentration is a consequence of the higher number of thinner bundles of EC polymers, which leads to increased network entanglement with an important contribution to the firmness of the oleogel.

On the other hand, the gelation mechanism of EC involves polymer–polymer interactions through the formation of hydrogen bonds between EC strands [3]. A higher concentration of EC will increase the number of hydrogen bonds, resulting in a robust network. The use of PO or RO also induced significant differences in the firmness and adhesiveness of the resulting oleogels. P-OG was the firmest gel, presenting hardness values ranging between 11.45 ± 0.09 N for P-OG7 and 14.02 ± 0.18 N for P-OG9. In contrast, RO-based oleogels were notably softer, with hardness values ranging between 0.20 ± 0.04 N for R-OG7 and 3.53 ± 0.25 N for R-OG9. The combination of PO and RO in various combinations induced intermediate hardness values for obtained oleogels, depending on the mixing ratios. RP-OG7 and RP-OG9, where rapeseed oil was higher (PO:RO 1:1), exhibited a lower hardness than the oleogels obtained from uncombined oils P-OG and R-OG, regardless of EC concentration. The oleogels based on the PO:RO 3:1 combination showed a higher value than those that were RO-based but lower than those based on PO, regardless of the oleogelator concentration. These findings agree with previous studies showing that the influence of oil type is attributed to its composition. Oils rich in highly polyunsaturated fatty acids (PUFAs) are shown to produce firmer gels [9,29]. The hardness of oleogel samples with high PO content was closely correlated with their high PUFA content, which allowed more interactions between polymer chains, increasing the gels’ structural stability and firmness. PO, with a content of 42–55.9 g PUFA/100 g oil [60,61], generated oleogels with higher firmness than oleogels based on RO, whose content in PUFA is 20.73–29.6 g/100 g oil [62], which indicated a more robust network between PO and EC compared to RO and EC. The same trend was observed for adhesiveness values, where the PO oleogels were the most adhesive, and the RO samples showed the lowest values.

## 3. Conclusions

The present study aims to explore the potential of PO and RO, alone or in various combinations, to obtain EC oleogels as possible animal fat substitutes in food products. Oleogels were obtained by dispersing EC in oil preheated to 155 °C, cooling to room temperature and storing at 4 °C.

High-OBC oleogels were obtained for most of the samples. The FTIR spectra showed a similar binding behavior of the oils in a stable three-dimensional network with different EC concentrations. In terms of texture, PO produced strong and stable oleogels. The mixture of PO with RO caused a significant reduction in the mechanical properties of the oleogels, while the RP-OG7 and R-OG7 samples did not gel. Regardless of oil type and EC concentration, all oleogels showed high oxidative stability after 14 days of storage, within limits provided by international standards for edible fats. Although PO and RO have a high content of unsaturated fatty acids susceptible to oxidative degradation, the low values of PV and TBARS for the first part of storage suggested their oxidative stability. However, further studies are needed to understand the behavior of fatty acids during oleogel preparation and storage.

Therefore, we can conclude that EC is an effective structuring agent for edible oils, but the characteristics of oleogels are strongly affected by oleogelator concentration and solvent type. The PO-predominant oleogels (P-OG and PR-OG) with a 9% EC concentration demonstrated the best characteristics when considering texture, GFT, OBC, and oxidative stability for further intended purposes. Thus, these oleogels are the most suitable for replacing saturated fats in food products. Future work will focus on replacing animal fat with the EC-based oleogels presented in this study, to obtain food products with increased nutritional value and acceptable organoleptic characteristics as a healthier alternative for consumers.

## 4. Materials and Methods

### 4.1. Materials

Cold-pressed pumpkin seed oil (PO) and refined rapeseed oil (RO) were obtained from local markets in Arad, Romania. EC, with an average viscosity of 64 cP, was purchased from Carl ROTH (Karlsruhe, Germany). Analytical grade chemicals, including acetic acid, chloroform, potassium iodide, sodium thiosulfate, trichloroacetic acid, thiobarbituric acid, and 1,1,3,3-tetra-methoxypropane were bought from Sigma-Aldrich Ltd. (Steinheim, Germany) or Merck, (Darmstadt, Germany).

### 4.2. Oleogels Preparation

**Figure 8:**
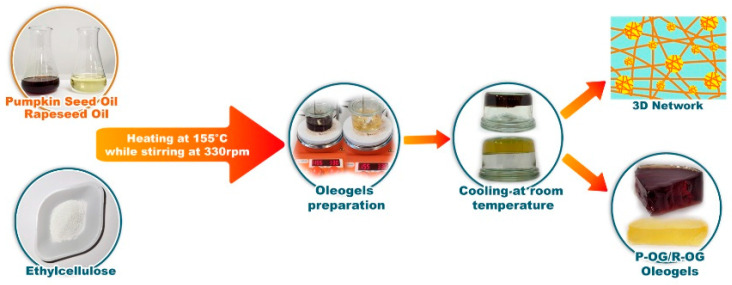
Schematic representation of oleogel preparation.

The method described by Silva et al. [33] was applied with a few modifications to prepare oleogels. Figure 8 presents a schematic representation of oleogel preparation.

Samples were obtained using two different EC concentrations, namely 7% (OG7) and 9% (OG9), while PO and RO were used both alone and in various combinations, as shown in Table 3. EC was directly dispersed in the oil and heated at 155 °C under continuous stirring at 330 rpm for 30 min until completely dissolved. Afterwards, the oleogels were cooled at room temperature (RT) (21 ± 3 °C) for 24 h and stored at 4 °C until further determinations were performed.

### 4.3. Visual Appearance

The method described by Fayaz et al. [63] was adapted for visual appearance analysis. After preparation, the oleogels were transferred into 100 mL containers and kept at RT until solidification. Subsequently, the containers were inverted to assess their stability, focusing on detecting any indications of phase separation or liquid oil on the surface.

### 4.4. Gel Formation Time

The gel formation time (GFT) analysis focused on determining the time taken for an oleogel to solidify in controlled conditions. Oleogel samples were placed into glass tubes and heated at 90 °C in a water bath for 1 h until fluidization. After that, the gelation process was monitored at RT over time. The duration of gelation was measured and recorded in minutes. To thoroughly observe the gelation process, the tubes were rotated at a 180° angle to monitor the flow carefully [64].

### 4.5. Oil-Binding Capacity

The oil-binding capacity (OBC) of the oleogel samples was ascertained using Zheng et al.s’ method [4]. After preparation, 15 g of oleogel was accurately weighed and added into tarred centrifugal tubes, and complete gelation was obtained after 24 h at room temperature. The tubes were weighted and centrifuged at 10.000 rotations/minute for 15 min. The tubes were then inverted for 30 min, the liquid oil was drained, and the tubes were reweighed. The gravimetric calculation of the OBC was based on the following Equation (1) [7]:

OBC (%) = [1 − (m_1_ − m_2_)/m_1_] × 100, (1)

where m_1_ and m_2_ are the masses of the samples before and after centrifugation.

### 4.6. Attenuated Total Reflectance-Fourier Transform Infrared

The attenuated total reflectance–Fourier transform infrared (ATR-FTIR) spectra of oils, ethylcellulose, and oleogel samples were recorded at room temperature as absorbance values within the wavelengths between 600 and 4000 cm^−1^, using a Bruker Vertex 70 Spectrometer (Bremen, Germany) equipped with a Pike Miracle ATR device. For ethylcellulose and oleogels, the samples were directly positioned onto the ZnSe ATR crystal surface and compressed using the device’s upper handle [40]. Oil samples’ spectra were obtained by placing equal volumes of 10 µL in the Teflon depression of the Pike Miracle ATR device. Spectrometric data were collected with a 4 cm^−1^ resolution. All samples underwent triplicate measurements, and an average spectrum was computed from three sets of 32 scans per sample [33]. Isopropyl alcohol was used to clean the ZnSe crystal before each ATR measurement, and an air background spectrum was captured. Data acquisition, normalization, and baseline correction were performed using OPUS 6.5 software.

### 4.7. Oxidative Stability

To ascertain the oxidative stability after processing (24 h) and 21 days of storage at accelerated oxidation conditions (60 °C), the lipid oxidation of oleogel samples was assessed [65]. After one, seven, fourteen, and twenty-one days, the degree of primary and secondary oxidation components was measured using the peroxide value (PV) and thiobarbituric acid reactive substances (TBARS). The results for fresh oils, which were stored under the same conditions as the oleogels, were compared to the oxidative stability of the oleogel samples. The oils included pumpkin seed oil (PO), rapeseed oil (RO), a 3:1 mixture of pumpkin seed oil and rapeseed oil (PRO), and a 1:1 mixture of pumpkin seed oil and rapeseed oil (RPO).

#### 4.7.1. Peroxide Value

The iodometric titration method developed by Dimakopoulou-Papazoglou et al. [66] was used to determine the PV of the oils and oleogel samples. In a flask, 5 g of each sample was dissolved by shaking in 30 mL acetic acid/chloroform (3:1) mix. After dissolution, 0.5 mL of saturated potassium iodide was added, and then the mixture was homogenized and kept for 15 min in the dark. At the end of the period, 1 mL of starch solution (1%) and 20 mL of distilled water were added to the mixture, and the resulting solution was titrated with sodium thiosulfate solution (0.01 N) until the color became transparent. The PV was calculated using Equation (2) [5] and expressed as milliequivalents of active oxygen per kilogram of oil (meq O_2_/kg):

PV = (V × N × 1000)/W,(2)

where V (mL) is the sodium thiosulfate volume, W is the sample weight (g), and N is the sodium thiosulfate solution normality.

#### 4.7.2. TBARS

The TBARS were determined according to the method described by Pan et al. [67], with some modifications. 0.5 g of oleogel was finely homogenized and mixed with 8 mL of TBARS reagent (15 g trichloroacetic acid, 0.375 g thiobarbituric acid, and 100 mL 0.25 M hydrochloric acid) until dissolution. The mixture was placed in a water bath at 100 °C for 15 min, immediately cooled down at room temperature and centrifuged at 8000 rpm/20 min using a Rotina 380R (Hettich, Germany) centrifuge. The lower phase was collected, and the Shimadzu UV-2250 spectrophotometer (Shimadzu Inc., Tokyo, Japan) was set at 532 nm for the absorbance determination. The results were calculated using 1,1,3,3-tetra-methoxypropane as a standard and expressed as mg malonaldehyde (MDA)/kg oil [68].

### 4.8. Texture Analysis

The texture of the oleogels was measured using a Texture Analyzer TX-700 (Lamy Rheology Instruments, Champagne au Mont d’Or, France) equipped with a 50 N load cell and a 12.7 mm diameter stainless plunger. Immediately after obtaining the oleogel samples, they were placed in polypropylene containers (4 cm inner diameter and 6.3 cm height) and, after gelation at room temperature, were cooled to 4 °C for 24 h. Oleogels’ texture was evaluated using a direct compression–relaxation–traction cycle under the following conditions: compression speed 1 mm/s; relaxation time 10 s; penetration depth 6 mm; and lifting speed after relaxation 1 mm/s [69,70]. The maximum force in the final position of the compression stage, the equilibrium force at the end of the relaxation period, and the retention force when lifting the probe were recorded. The firmness and adhesiveness of the oleogel samples were evaluated using Rheo Tex software. Firmness is the maximum force measured during penetration, while adhesiveness represents the opposing force necessary to pull the plunger away from the sample [7,71].

### 4.9. Statistical Analysis

Each determination was made three times. The findings are shown as mean values ± standard deviation (SD). Using the Excel Data Analysis Tool (Microsoft Office Professional Plus 2019), an analysis of variance (ANOVA) and Tukey’s multiple comparison test were carried out for statistical analysis, with a 95% confidence level.

## Figures and Tables

**Table 1:** Gel formation time for various EC oleogels.

Samples	GFT (min)
P-OG7	$3.10\;\pm \;0.18\;\begin{array}{c} b\\ 4 \end{array}$
PR-OG7	$3.50\;\pm \;0.18_{\;}\begin{array}{c} c\\ 4 \end{array}$
RP-OG7	no gel formation
R-OG7	no gel formation
P-OG9	$2.00\;\pm \;0.09_{\;}\begin{array}{c} a\\ 1 \end{array}$
PR-OG9	$2.15\;\pm \;0.13_{\;}\begin{array}{c} a\\ 1,2 \end{array}$
RP-OG9	$2.35\;\pm \;0.10_{\;}\begin{array}{c} a\\ 2,3 \end{array}$
R-OG9	$2.20\;\pm \;0.13_{\;}\begin{array}{c} a\\ 1,3 \end{array}$

Results are presented as mean ± SD. Different letters indicate significant differences within oleogel samples (*p* < 0.05). Different numbers indicate significant differences within oleogel samples with the same EC concentration (*p* < 0.05).

**Table 2:** Texture parameters for various EC oleogels.

Samples	Firmness (N)	Adhesiveness (N)
P-OG7	11.45 ± 0.09 $\begin{array}{c} e\\ 3 \end{array}$	−0.72 ± 0.01 $\begin{array}{c} f\\ 4 \end{array}$
PR-OG7	1.63 ± 0.04 $\begin{array}{c} b\\ 2 \end{array}$	−0.37 ± 0.02 $\begin{array}{c} d\\ 3 \end{array}$
RP-OG7	0.13 ± 0.04 $\begin{array}{c} a\\ 1 \end{array}$	−0.01 ± 0.00 $\begin{array}{c} a\\ 1 \end{array}$
R-OG7	0.20 ± 0.04 $\begin{array}{c} a\\ 1 \end{array}$	−0.06 ± 0.01 $\begin{array}{c} b\\ 2 \end{array}$
P-OG9	14.02 ± 0.18 $\begin{array}{c} f\\ 8 \end{array}$	−0.95 ± 0.03 $\begin{array}{c} g\\ 7 \end{array}$
PR-OG9	5.46 ± 0.10 $\begin{array}{c} d\\ 7 \end{array}$	−0.48 ± 0.01 $\begin{array}{c} e\\ 6 \end{array}$
RP-OG9	1.37 ± 0.02 $\begin{array}{c} b\\ 5 \end{array}$	−0.18 ± 0.01 $\begin{array}{c} c\\ 5 \end{array}$
R-OG9	3.53 ± 0.25 $\begin{array}{c} c\\ 6 \end{array}$	−0.15 ± 0.02 $\begin{array}{c} c\\ 5 \end{array}$

Results are presented as mean ± SD. Different letters in the same column indicate significant differences within oleogel samples (*p* < 0.05). 1–4 Different numbers in the same column indicate significant differences within oleogel samples with 7% EC (OG7) (*p* < 0.05); 5–8 Different numbers in the same column indicate significant differences within oleogel samples with 9% EC (OG9) (*p* < 0.05).

**Table 3:** Oleogel samples’ codification and composition.

Sample Codification	EC (%)	PO (%)	RO (%)
P-OG7	7	100	0
PR-OG7	7	75	25
RP-OG7	7	50	50
R-OG7	7	0	100
P-OG9	9	100	0
PR-OG9	9	75	25
RP-OG9	9	50	50
R-OG9	9	0	100

## Data Availability

The data presented in this study are openly available in article.
